# A 9-year experience study of single-port micro-laparoscopic repair of pediatric inguinal hernia using a simple needle

**DOI:** 10.1007/s10029-019-02079-4

**Published:** 2019-12-31

**Authors:** R. Chen, S. Tang, Q. Lu, X. Zhang, W. Zhang, Z. Chen, S. Qi

**Affiliations:** Department of General Surgery, Tungwah Hospital Affiliated With Sun Yat-Sen University, Dongguan, 523110 Guangdong China

**Keywords:** Inguinal hernia, Micro-laparoscopic, Single-port, Children

## Abstract

**Purpose:**

As laparoscopic techniques and equipments improve, laparoscopic inguinal hernia repair has been gaining popularity. The objective of the study was to summarize 9 years of experience using a single-port micro-laparoscopic approach to repair pediatric inguinal hernias with a simple hernia needle.

**Methods:**

1880 children with inguinal hernias were enrolled using micro-laparoscopic surgery between June 2009 and 2018. All patients underwent high ligation surgery using a single-port micro-laparoscopic technique. The clinical data were retrospectively analyzed.

**Results:**

All micro-laparoscopic surgeries were successfully performed in the 1880 patients, who ranged in age from 2 months to 14 years (3.66 ± 2.96 years) including 1622 males and 258 females. Among them, 1299 cases were unilateral hernias and 581 cases were bilateral hernias. The average operating time was 12.5 ± 3.5 min for a unilateral hernia and 20.5 ± 4.5 min for bilateral hernias. All patients were discharged 1–2 days after surgery, and the average length of their hospital stay was 2–4 days. Complications of knot reaction and pneumoscrotum occurred in 5 cases (0.27%) and 54 cases (2.87%), respectively, but these cases were properly managed, with no major impact on the operational outcomes. All patients were followed up for 3–65 months; there were 13 recurrent cases (0.69%).

**Conclusions:**

Single-port micro-laparoscopic herniorrhaphy in children using a simple hernia needle is a reliable and minimally invasive procedure.

## Introduction

In children, inguinal hernia repair requires only the high ligation of the hernia sac. Traditional open pediatric herniorrhaphy entails performing an inguinal incision and dissecting the inguinal canal, but using this method can easily destroy the normal anatomy of inguinal canal. The traditional approach leaves behind relatively greater damage and may result in many complications. In addition, a contralateral occult hernia may be misdiagnosed. With the development of laparoscopic surgical techniques, laparoscopic inguinal hernia repair can avoid these shortcomings and allow the high ligation of the hernia sac, which can reduce the recurrence rate [1].

The laparoscopic ligation of the hernia sac has been widely applied in clinical practice, and various methods have been developed by different teams [2, 3]. They may exit some pitfalls, such as complicated procedure and special equipments; in addition, a large number of clinical cases of a single-port laparoscopic inguinal hernia repair are rarely reported. Since June 2009, 1880 pediatric inguinal hernia cases have been treated using single-port micro-laparoscopic herniorrhaphy, and good outcomes were achieved. In this study, we described the surgical procedure and reported the patients’ outcomes.

## General information

1880 cases were enrolled for the study. They were all confirmed the diagnosis of inguinal hernia by symptoms, signs and ultrasonography performed before operation. Cases of irreducible and incarcerated hernias were excluded.

## Materials and methods

**Fig.1:**
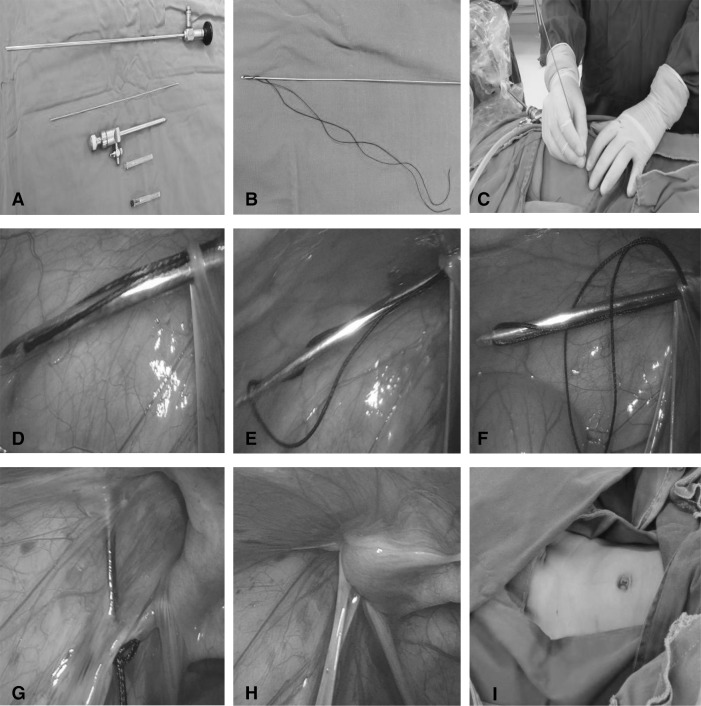
The equipment, materials and procedure of the surgery. **a** The equipment and materials. **b** The hernia needle with a non-absorbable silk suture. **c** The specialist was operating. **d** The needle inserted into abdominal cavity. **e** The needle with suture-1 withdrawn and left suture loop. **f** The needle with suture-2 inserted into suture-1 loop. **g** The suture-2 was brought out to encircle the internal ring. **h** The internal ring after high ligation. **i** After surgery, the incision was hardly seen

The required equipments and materials for our procedure were as follows (Fig. 1a): a Wolf or Olympus laparoscopic system, a 30° pediatric laparoscopic lens 5 mm in diameter, a trocar 5 mm in diameter, a hernia needle 1.3–1.5 mm in diameter with a hole in one flat, blunt terminal. This could be a processed Kirschner pin with a hole in the flattened and blunted terminal, threaded with a size 4 or 7 non-absorbable silk suture (Fig. 1b), a size 7 needle for pinpoint, and a size 12 needle for incision.

**Fig. 2:**
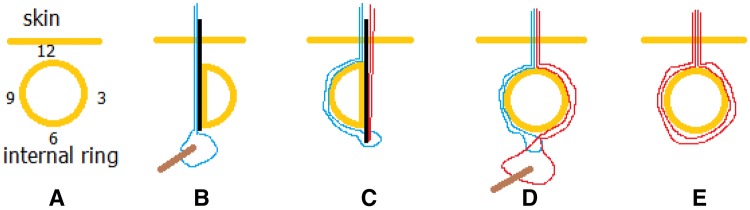
Schematic diagram of high ligation. **a** Internal ring. **b** The laparoscope was inserted into the suture-1 loop. **c** The needle with suture-2 inserted into the suture-1 loop.** d** the laparoscope was inserted into the suture-2 loop. **e** The suture-2 was brought out by suture-1

Surgical procedure (Figs. 1c–i, 2a–e): after the induction of general anesthesia, patients were placed in the supine position, with hips elevated. A 5 mm port was created in the umbilical region for the insertion of a 5 mm laparoscope and pneumoperitoneum was maintained at 6–10 mmHg. The presence of the hernia was confirmed through the examination of the internal inguinal ring. A size 7 needle was inserted at the point of the internal inguinal ring to locate the exact puncture position; then, a size 12 needle was used to increase the incision by 1–2 mm. A hernia needle threaded with a size 4 or 7 suture was inserted into the incision at the 12 o’clock position extraperitoneally, very close to the peritoneum on the medial side of the inguinal ring. The peritoneum was then punctured at the 6 or 7 o’clock position. The needle was then advanced intraperitoneally to the upper-right region of the abdomen for a right-sided hernia, or the upper-left region of the abdomen for a left-sided hernia. The laparoscope was inserted into the suture loop and advanced to the lateral abdominal wall. Thereafter, the needle was withdrawn and the suture loop remained in the abdominal cavity. Next, in the same incision, the needle with a size 4 or 7 suture was inserted into the lateral side of the inguinal ring, where it punctured the peritoneum at the 6 or 7 o’clock position—the original needle’s outlet—and entered the loop. Using the same method, the second needle was withdrawn, leaving the second suture loop in the abdominal cavity; the ends of the first suture was then pulled out to bring out the second suture loop. The second suture loop was cut, and a knot was tied subcutaneously. Either double-suture high ligation or single-suture high ligation was accomplished. Care should be taken not to ligate the vas deferens, spermatic vessels, epigastric artery, or iliac vessels during the procedure. The same procedure was performed on the contralateral side for patients with bilateral hernias. To conclude the procedure, the pneumoperitoneum was relieved, the trocar was removed, and the mini-incisions were closed using skin glue.

## Results

### Patients

1880 cases were treated. They were 2 months to 14 years of age, including 1622 males and 258 females. The male-to-female ratio was 6:1. There were 1299 cases with a unilateral hernia (740 right-sided and 559 left-sided hernias) and 581 cases with bilateral hernias. Contralateral occult hernias occurred in 77 patients initially diagnosed with unilateral hernias, as shown in Table 1.

**Table 1:** Patients’ characteristics (*n* = 1880)

Parameters	Observations
No. of cases	1880
Mean age	3.66 ± 2.96 years (2 months to 14 years)
Sex	
Male	1622 (86%)
Female	258 (14%)
Side of hernia	
Right	740 (39%)
Left	559 (30%)
Bilateral	581 (31%)
Contralateral occult	77 (4%)
Mean operative time	12.5 ± 3.5 min (unilateral)
	20.5 ± 4.5 min (bilateral)
Follow-up	3 ~ 65 months
Hospital stay	3.76 ± 1.05 days
Complications	
Knot reaction	5 (0.27%)
Pneumoscrotum	54 (2.87%)
Recurrence	13 (0.69%)

### Operation

The average operation time for micro-laparoscopic high ligation of the hernia sac was 12.5 ± 3.5 min for a unilateral hernias and 20.5 ± 4.5 min for bilateral hernias. All patients were discharged 1–2 days after surgery, and the hospital stay lasted 2–4 days, and the average hospital stay was 3.76 ± 1.05 days.

### Postoperative complications

There were no complications in majority patients, except for a tissue reaction and pneumoscrotum occurred in 59 patients. The tissue reaction surrounding the surgical knots occurred in 5 cases (0.27%). Two of these cases healed naturally, two healed after the sutures were removed without recurrence, but one recurrent after the suture was removed healed by open surgery. Pneumoscrotum occurred in 54 cases (2.87%) after surgery, and all were absorbed within one month. No other complications occurred, such as infection of the incision, seroma, bleeding, adhesive intestinal obstruction, iatrogenic cryptorchidism.

All patients were followed up for 3–65 months, and there were 13 recurrent cases (0.69%).

## Discussion

Pediatric inguinal hernias are common in pediatric surgery with an occurrence rate of about 0.8–4.4% [4]. These types of hernias often occur in children less than one year of age, especially within a few months after birth, and the male-to-female ratio is about 15:1. About 60% of inguinal hernias occur on the right side, while bilateral hernias account for about 10% of all cases [5, 6]. In our study, the male-to-female ratio was about 6:1, in which the trend of sex ratio was consistent with the reports. Male susceptible is concerned with gender anatomy.

In contrast with adult inguinal hernias, pediatric inguinal hernias typically result from the processus vaginalis’s failure to close during fetal development. Weak muscles in the groin area are not the main cause of pediatric inguinal hernias, and any weak muscles present can be improved during the fetus’s development. In addition, high ligation of the hernia sac alone may cure pediatric inguinal hernias [7]. To achieve high ligation of the hernia sac in a traditional open procedure, the preperitoneal structure must be dissected, which may undermine the anatomy of the inguinal canal and injure the vas deferens, spermatic vessels, nerves, cremaster, among other things. The location of the inguinal inner ring is close to the anterior abdominal wall and can easily be exposed by laparoscope. Therefore, laparoscopic treatment is feasible and less invasive than an open repair.

Laparoscopic treatment has several advantages: there is no need to dissect the inguinal canal, or the spermatic cord; it is easier to locate the hernia sac, damaging neither the cremaster nor the vas deferens and without having to incise the hernia sac; a reliable location is provided for inner ring high ligation; and the closure of the inner ring after ligation can be easily observed. In addition, any occult contralateral inguinal hernias can quickly be identified and repaired during the same surgery to avoid the need for a subsequent procedure. Laparoscopic high ligation is an ultra-high-bit hernia sac ligation, and the recurrence rate is reduced when compared against traditional surgery [8–10]. However, some studies have reported that the recurrence rate is not different between laparoscopic high ligation and traditional open surgery [11]. Due to the weak abdominal muscles and abdominal walls of children, the use of a low-pressure pneumoperitoneum could ensure a sufficient surgical field. Due to the short operation time, low-pressure CO_2_ pneumoperitoneum will not seriously affect the respiration, circulation, or CO_2_ retention in children [12, 13]. During the surgical procedure, the instruments do not directly touch the bowel or omentum majus, and the incision in the umbilical region is small, which significantly reduces the possibility of complications, such as adhesions.

Most reported laparoscopic inguinal hernia surgeries use a two-port method [14, 15]; a single-port approach is rarely applied [16, 17]. The two-port method is more complicated and often causes greater trauma than the single-port technique. Due to the small amount of space in the pediatric abdomen, it is difficult for the instruments to enter the abdomen through the second port. In the single-port approach, special surgical instruments are required, but the operative procedure is quite simple and can achieve a similar outcome as the two-port method with minimal trauma. The reported surgical techniques and instruments are quite complicated [16–18]. We modified the single-port approach using an easily available puncture needle to retrieve the second thread loop. This procedure is simple and is worth applying in clinical practice.

These modified methods offer the following advantages: (1) minimal invasiveness: the surgical incision required for a traditional open repair is commonly 3–5 cm; in contrast, the incision needed for the modified procedure is 1–2 mm in the groin area and 0.5 cm in the umbilical region. No wound suturing is necessary in this updated surgery; the use of biogel to close the wound prevents scarring. Compared with the two- or three-port laparoscopic approach, this procedure calls for a 0.5 cm incision to be made in the umbilical region, which achieves a similar effect as surgery, but with minimal trauma. Compared with the use of a crochet hook, which may damage the spermatic vessels or vessels on the abdominal wall and lead to extraperitoneal hematoma, the use of a simple hernia needle about 1.5 mm in diameter may also decrease the amount of trauma. (2) Rapid recovery: this surgery does not involve the groin area, and only a small incision is made, which allows for patients to be discharged within 1–2 days following the surgery, and to recover in a short period of time. (3) Low recurrence and complication rates: in contrast with the traditional open surgery, laparoscopic surgery involves no dissection of the groin area. The inguinal canal and the spermatic cord are viewed directly, leading to a low occurrence of complications like inguinal or scrotal edema, subcutaneous hydrops, postoperative bleeding, iatrogenic cryptorchidism, and infection of incisions, among other things. Moreover, the 0.69% recurrence rate for this modified procedure is significantly lower than that of the traditional open surgery, which is 2–6.3% [19]. (4) Simplicity of the operation: in the open procedure, surgeons must take care to avoid injuring the cremaster, spermatic cord, blood vessels, nerves, and other structures during their dissection. However, in this modified surgery, only the vas deferens, spermatic vessels, inferior epigastric artery, and iliac vessels require avoidance. In addition, the surgical time is reduced significantly. The average operative time of this surgery for a unilateral hernia is 12 min, and bilateral hernias require 20 min. (5) Contralateral examination: cases with a dominant hernia on one side and an occult hernia on the other side do not have to undergo further procedures, since the laparoscope can be used to visualize both sides and identify an occult hernia. In pediatric patients, the incidence of occult hernia is 20.0–39.7% [20–22]. In our study, contralateral occult hernias were present in 77 cases (or 4.10%); if these patients had undergone a traditional open repair, they would have eventually required a second operation to repair the occult hernia.

Matters which should be paid attention during operation include: the abdominal wall in children is thin, and towel clamps should not be used, to avoid accidentally damaging organs. The trocar should be inserted using rotary force, and violent pushing should be avoided. A size 7 needle should be used to determine the exact location of the internal ring, the suture we use is non-absorbable surgical silk suture. During the operation, it is necessary to avoid the blood vessels under the abdominal wall, as well as the spermatic vessels and vas deferens; these structures should not be damaged or ligated. A simple method for the avoidance of these areas is to move the puncture needle along the vas deferens to the pelvic sidewall, where the adhesion between the peritoneum and the vas deferens is loose. In this study, 581 bilateral hernia cases received high ligation on both sides, and all recovered very well after surgery. The hospital stay for patients treated for bilateral hernias was similar to that of unilateral hernia cases, relatively reducing the overall cost, since a second surgery on the opposite side was avoided. Besides the laparoscope, no other instruments, such as forceps or a needle holder, were required to enter the abdominal cavity, thus reducing the unnecessary usage of surgical instruments, shortening the operation time, and reducing the amount of trauma.

Postoperative complication of pneumoscrotum occurred in 54 cases in the early stage of our operation due to a lack of experience. After surgical procedure optimized, there was no such complication occurred. In our experience, before tying the knot in the thread, the scrotum should be squeezed to expel any gas in the hernia sac, thereby avoiding postoperative pneumatosis.

There were five cases of knot reaction in the early stage of our operation, two of them healed naturally, two healed after the sutures were removed without recurrence, but one recurrent after the suture was removed healed by open surgery. In our experience, after tying the suture knot, the skin at the knot should be lifted to place the knot under the skin without knot exposure, to avoid a skin reaction in the area surrounding the knot.

Thirteen of the patients experienced a recurrence, with possible reasons including: (1) the patients were quite young, and the abdominal wall was relatively lax and thin; (2) a large hernia ring, a recent history of an incarcerated oblique hernia, or local edema; (3) loose or broken thread; (4) the use of absorbable thread; and (5) surgical errors due to a lack of experience.

## Conclusions

In the study, we reported 9 years experience on 1880 children with inguinal hernias undergoing micro-laparoscopic herniorrhaphy using a simple hernia needle. This technique we applied received satisfactory results; it had the advantages of being minimally invasive, having a quick recovery time with a low recurrence rate, and being cosmetically appealing. Therefore, the single-port approach should be widely applied for pediatric inguinal hernia treatment.
